# Whole Transcriptome Analysis Reveals Heterogeneity in B Cell Memory Populations in Patients With Juvenile Idiopathic Arthritis-Associated Uveitis

**DOI:** 10.3389/fimmu.2020.02170

**Published:** 2020-09-17

**Authors:** Roos A. W. Wennink, Aridaman Pandit, Anne-Mieke J. W. Haasnoot, Sanne Hiddingh, Viera Kalinina Ayuso, Nico M. Wulffraat, Bas J. Vastert, Timothy R. D. J. Radstake, Joke H. de Boer, Jonas J. W. Kuiper

**Affiliations:** ^1^Department of Ophthalmology, University Medical Center Utrecht, Utrecht University, Utrecht, Netherlands; ^2^Laboratory of Translational Immunology, University Medical Center Utrecht, Utrecht University, Utrecht, Netherlands; ^3^Department of Pediatric Rheumatology, University Medical Center Utrecht, Utrecht, Netherlands; ^4^Department of Rheumatology and Clinical Immunology, University Medical Center Utrecht, Utrecht, Netherlands

**Keywords:** juvenile idiopathic arthritis, juvenile idiopathic arthritis associated uveitis, RNA sequencing, B cell, memory B cell

## Abstract

**Purpose:**

Patients with juvenile idiopathic arthritis (JIA) are prone to developing chronic anterior uveitis (JIA-U+). Although several risk factors for JIA-U+ have been identified, the underlying etiology is poorly understood. Histopathological studies demonstrate B cell infiltrates in eye tissues of patients with JIA-U+.

**Methods:**

We performed transcriptome profiling of peripheral blood CD19-positive B cells taken from 14 cases with JIA-U+, 13 JIA cases without uveitis (JIA-U−), and five healthy controls. Deconvolution-based estimation was used to determine the immune cell fractions for each sample.

**Results:**

Deconvolution results revealed that naive B cells made up on average 71% of the CD19-positive cell fractions analyzed. Differential expression analysis identified 614 differentially expressed genes (DEGs) between the groups at nominal significance and six genes at a false discovery rate of 5% (FDR < 0.05). Head-to-head comparison of all JIA-U− versus JIA-U+ revealed no DEGs in the CD19+ B cell pool (FDR < 0.05). However, principal component analysis based on a panel of key genes for B cell subsets revealed that JIA-U+ cases bifurcate into distinct clusters, characterized by markedly disparate expression for genes associated with specific memory B cell populations. CIBERSORT analysis of the overall transcriptome of the new uveitis cluster identified an increased proportion of memory B cells.

**Conclusion:**

These data show that JIA-U− and JIA-U+ have a globally similar transcriptome considering the global peripheral CD19-positive B cell pool. However, heterogeneity in B cell memory genes among cases with uveitis suggests a role for specific memory B cell subsets in the etiology of JIA-U+.

## Introduction

Chronic anterior uveitis (CAU) is a common feature of oligo- and poly-articular juvenile idiopathic arthritis (JIA) that leads to unilaterally blindness in 6–24% of the children and 33% of the eyes have become visually impaired in adulthood (1–3). Although the incidence of uveitis in JIA varies between studies, approximately one third of JIA cases typically develop CAU within 4 years after JIA onset (4, 5). JIA-associated uveitis (JIA-U+) is often characterized by an insidious onset with a high risk for developing visually threatening complications. Consequently, JIA-U+ warrants intensive monitoring and requires specialized ophthalmological care.

It remains highly challenging to predict the onset of uveitis in advance. However, advances in clinical and molecular profiling studies have helped in better understanding which children are particularly prone to developing uveitis (6). A recent genome-wide association study highlighted distinct genetic susceptibility for uveitis in JIA (6, 7). JIA cases with uveitis more often are anti-nuclear antibody (ANA) positive and have increased levels of erythrocyte sedimentation rate (ESR) and S100A12 (calcium-binding protein) (6). Recently, flow cytometry studies have linked changes in blood T cells and monocytes to uveitis in JIA (8, 9). In contrast, immunohistochemical studies from iris biopsies and enucleated eyes revealed predominately infiltrating plasma cells and CD20-positive B cells (10, 11). The contribution of B cells in the pathophysiology of JIA-U+ is supported by the observation that anti-CD20 monoclonal antibody therapy (Rituximab) is effective in treating patients with (severe) JIA associated uveitis (12, 13). The association of ANA with uveitis in JIA also supports that B cell hyperactivity contributes to the development of uveitis in cases with JIA (6, 14, 15). Here, we report on the investigation of the transcriptome of peripheral blood B cells of cases with JIA and JIA-associated uveitis.

## Materials and Methods

### Patients and Patient Material

The study was approved by the Medical Ethical Research Committee in Utrecht in concordance with the Helsinki principles. Written informed consent was obtained from all patients if they were 18 years or older, from both parents and patients if they were 12–18 years of age and from parents only if they were younger than 12 years old.

We collected heparinized venous blood from a total of 32 children (≤16 year) with juvenile idiopathic arthritis with uveitis (JIA-U+, *n* = 14), and children with JIA without uveitis (JIA-U−, *n* = 13) and healthy controls (HC, *n* = 5) visiting the ophthalmologist or the pediatric rheumatologist at the University Medical Center Utrecht in the Netherlands. The JIA diagnosis was confirmed by a pediatric rheumatologist based on the criteria of the International League of Associations of Rheumatology (16). All patients were screened by an ophthalmologist specialized in childhood uveitis according to the guidelines of the Academy of Pediatrics (17). The patients with JIA-U− had an ophthalmologic follow-up of at least 4 years without signs of uveitis. JIA-associated uveitis was diagnosed according to the Standardization of Uveitis Nomenclature (SUN) criteria (18). All patients had active uveitis at time of sampling. None of the patients received immunomodulatory treatment other than methotrexate (Table 1). The Wilcoxon rank sum and Kruskal–Wallis test were used to assess group differences for continuous variables and Fisher’s Exact Test, Pearson’s Chi-square test for categorical variables. *P*-values below 0.05 were considered nominal significant.

**TABLE 1:** Characteristics of the cohort investigated in this study.

	**JIA**	**HC**	**JIA-U-**	**JIA-U+**	***P*-value**
*N*	27	5	13	14	NA
Male, *n* (%)	8	1 (20)	4 (31)	4 (29)	1.000
Age in years; median (IQR)	12 (9–19)	26 (26–27)	14 (10–19.5)	11 (8–19)	0.002
Methotrexate therapy, *n* (%)	16 (59%)	NA	6 (46)	10 (71)	0.182
ANA positivity, *n* (%)	20 (74%)	NA	7 (54)	13 (93)	0.033
Age at uveitis diagnosis; median (IQR)	6 (4–8)	NA	NA	6 (4–8)	NA
Age at arthritis diagnosis; median (IQR)	6 (2–9)	NA	8 (4.5–10)	2 (1–6.5)	0.005
Duration of uveitis in years; median (IQR)	5 (3–10)	NA	NA	5 (3–10)	NA

*HC, healthy controls; JIA-U−, juvenile idiopathic arthritis without uveitis; JIA-U+, juvenile idiopathic arthritis wit uveitis; ANA, anti-nuclear antibody; N.A., not applicable; IQR, interquartile range.*

### B Cell Isolation

For each case peripheral blood mononuclear cells (PBMCs) were isolated by standard ficoll gradient centrifugation from 9 mL heparinized blood immediately after blood withdrawal and subjected to sorting by The BD FACSAria^TM^ III sorter after incubation with antibody-conjugated surface antibodies (Supplementary Table S1) and FACS buffer (1% bovine serum albumin and 0.1% sodium azide in phosphate buffered saline) and blood lymphocytes purified (CD14^–^CD3^+^CD19^+^) peripheral (PBL) were stored in liquid nitrogen for later analysis. PBL samples were thawed in batches of 4–8 samples, divided over 5 days, washed with ice cold phosphate buffered saline and stained using the fluorescently conjugated antibodies in Supplementary Table S2. CD45^+^CD3^–^CD19^+^ cells were sorted using The BD FACSAria^TM^ III sorter. An example of the gating strategy is provided under Supplementary Figure S1.

### RNA Sequencing

FACS purified CD19^+^ B cells were immediately taken up in lysis buffer (RLT plus, Qiagen, Venlo, Netherlands) containing 1% β-mercaptoethanol and subjected to RNA extraction using the AllPrep Universal Kit (Qiagen) on the QIAcube according to the manufacturer’s instructions. In three samples the RNA was from insufficient quality and these samples were retained from further analysis. cDNA libraries were generated by GenomeScan (Leiden, Netherlands) with the TruSeq RNAseq RNA Library Prep Kit (Illumina Inc., Ipswich, MA, United States), and were sequenced using Illumina HiSeq 4000 generating ∼20 million 150 bp paired ended reads for each sample.

### Differential Gene Expression

FastQC tool was used for the quality check of the raw sequences. Reads were aligned to the human genome using STAR aligner (19) and Python package HTSeq (20) was used to count the number of reads overlapping each annotated gene. Count data were fed into DESeq2 (21) to conduct differentially expression analysis. Subsequently, DESeq2 was used to model the biological variability and overdispersion in the gene expression data as a negative binomial distribution. We used Wald’s test to identify DEGs in pairwise comparison and used likelihood ratio test (LRT) to identify DEGs considering multiple disease groups. We used variance stabilizing transformation (vsd) to plot normalized gene counts. We corrected *P*-values using a false discovery rate (FDR) of 5% according to the Benjamini and Hochberg method.

### Principal Component Analysis

We extracted expression data for 13 marker genes that distinguish peripheral blood B cell subsets as determined by single-cell sequencing analysis (22) and also include genes encoding IgA, IgM, and IgG (17 genes in total, see Supplementary Table S3), because previous studies have linked immunoglobulin expression to juvenile idiopathic arthritis-associated uveitis (23). Differences in the expression of these genes was considered to indicate changes in (memory) B cell subsets in blood, which would be evident by using this set of genes to conduct principal component analysis with the *factoextra* package in R. Using the first two principal components, we reclassified patients into new groups. We next used deconvolution-based estimation of memory B cell fractions (see below) and differential expression analysis by DESeq2 (21) to support if the PCA-identified clusters of patients were genuinely characterized by changes in gene expression. Expression data (median TPM values) for relevant B cell genes in purified blood naive B cells, non-switched B cells, and switched B cells as determined by RNA sequencing was obtained from Monaco et al. (24).

### Deconvolution-Based Estimation of Cell Fractions

For deconvolution of immune cell composition per patient sample, the gene expression data were analyzed by CIBERSORT using the standard reference signature expression matrix for leukocytes (LM22) (25). Kruskal–Wallis test was used to assess group differences for estimated B cell populations in cell fractions.

## Results

Patient characteristics are shown in Table 1. From a total of 27 patients with JIA, 20 patients (74.1%) were diagnosed with oligoarticular subtype, 6 patients with rheumatoid factor – negative polyarthritis (22.2%). Among the JIA patients with uveitis, 10 (71%) were classified with the oligoarticular subtype and 3 (21%) patients with rheumatoid factor-negative polyarthritis. As expected, patients with uveitis were more often ANA-positive and had a lower age of onset of JIA compared to patients without uveitis (Table 1).

**FIGURE 1:**
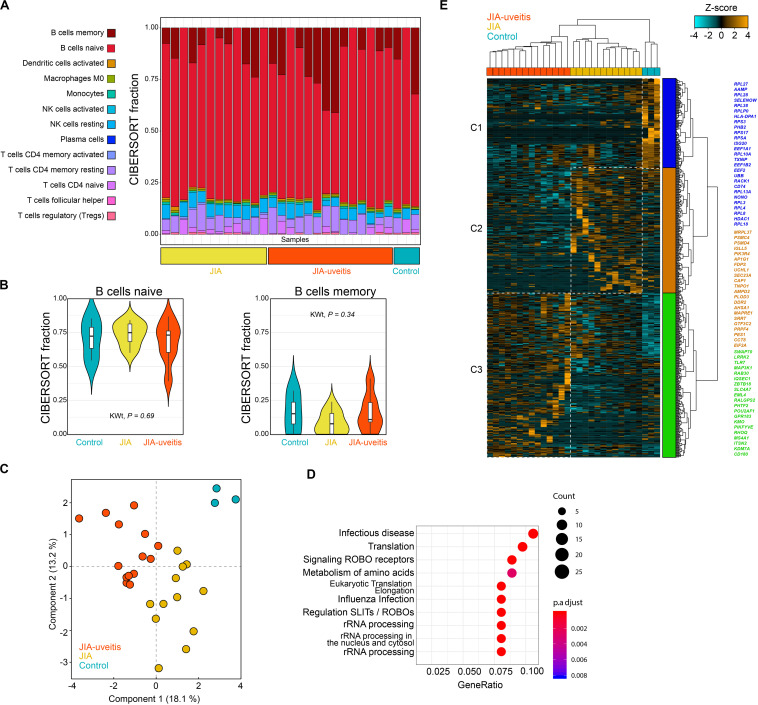
Blood CD19^+^ B cell transcriptomics in juvenile idiopathic arthritis-associated uveitis. **(A)** Deconvolution-based estimation of immune cell fractions using the RNA-sequencing data and CIBERSORT (25). The estimated cell fraction for each immune cell type in each sample is depicted in a stacked barplot (using the LM22 as signature sets to estimate the cell fractions) in the disease groups and controls. **(B)** The estimated cell fraction for naive and memory B cells for each of the disease groups and Kruskal–Wallis test statistic (KWt). **(C)** Principal component analysis of 614 DEGs at nominal significance (likelihood ratio test *P* < 0.05). **(D)** Pathway enrichment analysis of the 614 DEGs using the R package *clusterProfiler* with the *Reactome* database. **(E)** Hierarchical clustering of the 614 DEGs from the **(C)** using Euclidean distance and Ward’s method. Cluster 1–3 are indicated by different colors. A selection of genes associated to each cluster is indicated by the corresponding color (full list of genes and clusters are depicted in Supplementary Tables S4, S6).

We purified the blood (CD14^–^CD3^–^) CD19^+^ B cell population by flow cytometry from JIA cases with and without uveitis and controls, and performed whole-transcriptome RNA sequencing. After quality control, a total of 29 samples were used to investigate the transcriptomic signature of blood B cells. The CD19^+^ lymphocyte fraction comprises several B cell populations, including naive and memory B cells. Therefore, we first estimated the immune-cell composition from the bulk transcriptomic data using deconvolution-based estimation by CIBERSORT (25). As expected, the relative fraction of B cells was high (>80%) across all samples (Figure 1A). Naive B cells made up the majority of the fraction (mean across all samples 71%), followed by memory B cells (mean, 13%), and several other lymphocytes populations (Figure 1A). The estimated non-B cell populations most likely represents spill over caused by a subset of marker genes from the signature matrices also expressed in other lymphocytes and therefore display lower cell-specificity (26). Regardless, we observed no evidence for changes in the memory or naive B cell subsets between the disease groups (Kruskal–Wallis test, *P* = 0.34 and *P* = 0.69, Figure 1B).

Next, the transcriptomic data was subjected to differential expression analysis, which revealed 614 differentially expressed genes (DEGs) at nominal significance between the groups (likelihood-ratio test, *P* < 0.05) and six DEGs at FDR of 5% (Figure 1C and Supplementary Tables S4, S5). Results of the pathway enrichmen analysis for the 614 DEGs is shown in Figure 1D. Hierarchical cluster analysis of the 614 DEGs discerned three overarching clusters labeled C1 to C3 (Figure 1E). The gene signatures of each of the three clusters typically corresponded with one of the investigated disease groups. The first cluster (C1) contained mostly genes of the ribosomal machinery (i.e., *RPL* and *RPS* genes, Figure 1E) and was relatively high expressed in controls. This cluster also contained uveitis risk gene *HLA-DPB1*, which was decreased in JIA-U+ cases (Log_2_FC = −0.33, *P* = 0.016) and JIA cases (JIA-U-) compared to controls (Log_2_FC = −0.34, *P* = 0.013). The second cluster (C2) of genes was relatively higher expressed in JIA cases without uveitis and contained genes involved in cholesterol biosynthesis and mevalonate pathway (e.g., *MVD, FDPS, SEC23A*). Cluster 3 (C3) represented a gene signature associated with uveitis that includes core B cell genes, including the *MS4A1* gene (encoding CD20), Toll-Like Receptors (TLR) *TLR7* and *CD180* that regulates B cells responses, and *ITSN2* a key gene required for antibody formation in B cells (Supplementary Table S6).

### Bulk B Cell Transcriptome Reveals Heterogeneity in JIA Cases With Uveitis

**FIGURE 2:**
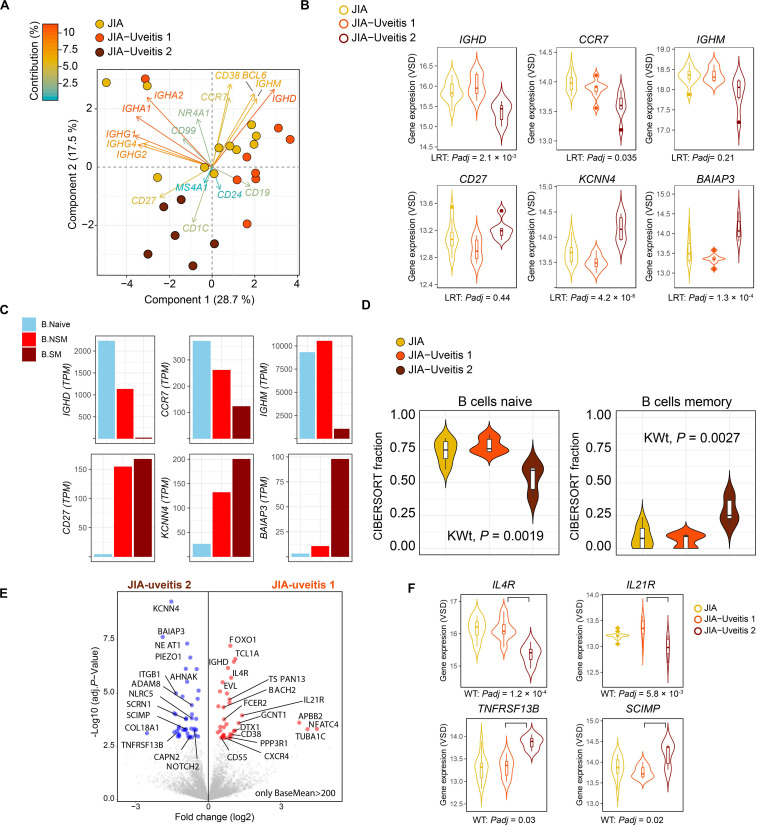
Heterogeneity in key B cell memory genes in JIA cases with uveitis. **(A)** Principal component analysis biplot of JIA cases with and without uveitis based on the expression data of 17 genes associated with B cell subsets. The contribution (in %) of each gene to the components is indicated. Two subgroup of uveitis cases are indicated in dark red and orange. **(B)** Gene expression for *CCR7*, *CD27, KCNN4, BAIAP3, IGHM*, and *IGHD* genes in JIA and the two JIA-uveitis clusters. LRT; likelihood ratio test from DESeq2 considering the JIA and the two JIA-uveitis clusters, Padj; FDR corrected *P*-values. **(C)** The median gene expression (TPM values) for genes of **(B)** in Naive B cells (B Naive), non-switched memory B cells (NS Mem), and switched memory B cells (S mem) from Monaco et al. (24). **(D)** Deconvolution-based estimation by CIBERSORT (25) of naive and memory B cells fraction for JIA and the two JIA-uveitis groups identified by the PCA analysis **(D)** under **(A)**. KWt, Kruskal–Wallis test. **(E)** Volcano plot of differentially expressed genes (DEG) from the head-to-head comparison of the JIA-uveitis cluster 1 versus JIA-uveitis cluster 2 **(A)**. The 10,859 high expressed genes (BaseMean >200 from DESeq2) are indicated in gray. DEGs are indicated in red (increased expression) and blue (decreased in expression). **(F)** Gene expression for *IL4R*, *IL21R, SCIMP*, and *TNFRSF13B* in JIA and the two JIA-uveitis clusters. WT, Wald’s test from DESeq2 considering head-to-head comparison of the newly identified two JIA-uveitis clusters, Padj, FDR corrected *P*-values.

Head-to-head comparison of JIA cases with and without uveitis revealed 387 DEGs at nominal significance (Supplementary Table S7) and no genes at a FDR of 5%. However, recent single cell analyses has revealed that the peripheral blood B cell compartment contains ∼10 functionally distinct B cell subsets (22, 27). Because the peripheral B cell fraction is dominated by naive B cells (Figure 1A), we hypothesized that B cell subset-specific gene expression from relatively rarer cell types in blood, may be drowned out in bulk transcriptome analysis. To this end, we assessed the relative expression of 17 marker genes previously associated with individual B cell subsets in peripheral blood mononuclear cells (Supplementary Table S3). Principal component analysis based on the panel of B cell subset genes revealed that three cases displayed relatively higher levels for Immunoglobulin G and A genes (Figure 2A). In addition, PCA analysis revealed heterogeneity among cases with uveitis. Overall, distinct clusters of uveitis cases (termed “JIA uveitis 2”) became apparent, characterized by distinct expression of *IGHD, CCR7, IGHM and CD27* (Figure 2B). Indeed, differential expression analysis based on the reclassification into three groups (JIA, JIA-uveitis 1, and JIA-uveitis 2, see Figure 2A) identified 41 genes at a FDR of 5%, including *IGHD* (LRT, *Padj* = 2.1 × 10^–3^) and *CCR7* (*Padj* = 3.7 × 10^–2^) and *KCNN4* (LRT, *Padj* = 4.2 × 10^–6^), and *BAIAP3* (LRT, *Padj* = 1.3 × 10^–4^) genes (Supplementary Table S8). Gene expression profiles for these genes associated with the distinct cluster of JIA-uveitis cases are reminiscent of the (IgD-, IgM-) switched memory B cells (Figure 2C) (24, 28). Gene set enrichment analysis revealed that genes associated with the reclassification of uveitis cases are associated with GO terms; *lymphocyte activation* (GO:0046649, *Padj* = 3.3 × 10^–5^), *B cell activation* (GO:0042113, *Padj* = 9.2 × 10^–4^), and *immune response-activating cell surface receptor signaling pathway* (GO:0002429, *Padj* = 9.1 × 10^–4^). To assess if the overall transcriptome of the newly identified uveitis cluster was indeed associated with memory B cells, we reanalyzed the B cell fractions using the signature genes of CIBERSORT (*n* = 547 genes). This analysis revealed that the identified subgroup of uveitis cases (JIA-uveitis 2 in Figure 2A) is characterized by a significantly increased proportion of memory B cells, coupled with a decrease in naive B cells compared to the other JIA groups (Kruskal–Wallis test; *P* = 0.0019, *P* = 0.0027, respectively, Figure 2D). Baseline characteristics of the distinct uveitis clusters are presented in Table 2.

**TABLE 2:** Characteristics of the two distinct uveitis clusters.

	**JIA uveitis 1**	**JIA uveitis 2**	***P*-value**
*N*	8	6	NA
Male, *n* (%)	3 (37.5)	1 (16.7)	0.58
Age in years; median (IQR)	9.5 (8–11)	20 (14–23)	0.003
Methotrexate therapy, *n* (%)	7 (87.5)	3 (50)	0.25
ANA positivity, *n* (%)	8 (100)	5 (83.3)	0.43
Age at uveitis diagnosis; median (IQR)	5.5 (3.5–7)	4.5 (8–12.50)	0.16
Age at arthritis diagnosis; median (IQR)	2 (1.25–7.4)	2 (1–5)	0.64
Duration of uveitis in years; median (IQR)	3 (2.25–4.75)	10 (5.75–15)	0.004
Duration of arthritis in years; median (IQR)	4.5 (2.25–7.5)	17 (6.75–21.25)	0.003
Biological use for uveitis, *n* (%)	4 (50)	5 (83.3)	0.31
Complications, *n* (%)	2 (25)	3 (50)	0.58

*ANA, anti-nuclear antibody; N.A., not applicable; IQR, interquartile range; Complications, cataract and glaucoma.*

Although age-related and disease course associated changes in memory B cells have been reported (29, 30) we did not observe a correlation between memory or naïve B cell population and age or disease duration (Supplementary Figure S2). We observed a relatively higher estimated memory B cell fraction in JIA cases with late-onset uveitis, but this difference was not statistically significant (*P* = 0.08) (Supplementary Figure S3).

Finally, head-to-head-comparison of the two newly identified JIA-uveitis clusters revealed 485 genes at a FDR of 5% (Supplementary Table S9). These included, as expected, *IGHD* (*Padj* = 9.4 × 10^–5^), *KCNN4* (*Padj* = 9.5 × 10^–8^), and *BAIAP3* (*Padj* = 2.2 × 10^–6^) (Figure 2E), but also revealed differences in the expression of key receptors implicated in B cell immunity such as cytokine receptors *IL4R* (*Padj* = 1.2 × 10^–4^), *IL21R* (*Padj* = 5.8 × 10^–3^), *TNFRSF13B* (*Padj* = 0.03), the receptor for B cell cytokines APRIL and BAFF, and *SCIMP* (*Padj* = 5.8 × 10^–3^), a transmembrane protein involved in major histocompatibility complex class II (i.e., HLA-DR) signaling (Figure 2F).

## Discussion

We performed transcriptome analysis of peripheral blood CD19^+^ B cells in JIA-U− and JIA-U+ patients. Our analysis suggests that the peripheral blood CD19^+^ B cells pool in JIA-U− and JIA-U+ patients display an overall remarkably similar transcriptome, also compared to controls. The differential expression analysis revealed only six differently expressed genes (DEGs) after FDR correction, which suggests that these differences are unlikely robust, but included genes such as *TXNIP* – related to B cell associated germinal centers in peripheral lymphoid organs (31) and *MN1*, linked to colony forming activity of B cells (32). Polymorphisms (SNPs) in the *HLA-DPB1* gene are associated with the susceptibility to uveitis in JIA (7), but the gene expression of *HLA-DPB1* was (slightly) decreased in JIA cases either with or without uveitis compared to controls (JIA-U+, Log2[FC] = −0.33, *P* = 0.016; JIA-U−, Log2[FC] = −0.34, *P* = 0.013). This can be explained by the fact that the variant associated with uveitis (rs6457109 in *HLA-DPB1*) is not an expression quantitative trait loci (33) for this gene, and thus, not associated with its expression, but predominantly with its peptide binding capacity.

We further show that, as expected, transcriptomic deconvolution revealed a large fraction of naive B cells in the total peripheral pool of CD19+ cells. This suggests that future analysis should focus on subsets beyond naive cells in JIA, for example using single cell RNA sequencing or high dimensional cytometry. Despite this limitation, this study may guide future studies into relevant B cell subsets. We exploited marker genes used to mark B cell subsets in peripheral blood mononuclear cells in recently reported single-cell studies (22, 27). We would like to emphasize that this set of genes is by no means exhaustive, but showed to be instrumental in our study to reclassify cases, because we showed that the newly identified subgroup of JIA-uveitis cases displayed genuine enrichment for memory B cell gene circuits as supported CIBERSORT analysis and distinct gene profiles as shown by differential expression analysis. Using this strategy, we identified a previously underappreciated heterogeneity among cases with uveitis that is characterized by increased proportion of memory B cells, most likely switched memory B cell populations. This is of interest, because in young patients with early onset oligoarticular JIA (a risk factor for uveitis) switched memory (CD27+ IgM-IgD-) B cells are expanded in blood and associated with the production of anti-nuclear antibodies (ANAs) (29). B-cell memory subsets that express relatively lower CCR7 are also increased in synovial fluid from oligoarticular JIA patients, suggesting that these cells also play a role in arthritis (34). However, a recent flow cytometry study revealed a decrease in naive B cells, coupled with an increase in switched B cells, and memory B cells in a subset of JIA patients (35). In light of the current study, these observations make it tempting to speculate that the changes in B cell composition in blood of JIA may in part be related to the presence or susceptibility to the development of uveitis. Considering uveitis as a multifactorial disease it is possibly mediated by redundant disease mechanisms. For example, increased circulating B cell memory may contribute significantly in a part of cases, while other factors may dominate in other cases. This is also reflected in clinical observations such as anti-nuclear antibodies, a risk factor for uveitis, that are more common – but not detected in all- uveitis cases. Although our study lacked sufficient power to investigate the association of the B cell signatures with clinical uveitis subgroups, genetic studies demonstrated that uveitis in JIA is genetically heterogeneous and may require reclassification into mechanistic disease endotypes (7).

Notably, however, post-switch IgD^–^CD27^+^ B cells increase with longer disease duration in a study with rheumatic arthritis patients (36). This of interest, because clinical data of patients in this study suggest comparable trends, with cases in JIA-Uveitis 2 showing longer disease duration compared to the other groups. In contrast, in B cell-mediated inflammatory conditions such as Sjogren’s disease longer disease duration is associated with a decrease in memory B cell subsets and a more active disease profile (37). Furthermore, although age-related changes in memory B cell subsets have been reported, the absolute number of peripheral blood naïve and memory B cell subsets both decline from ±2–4 years old until adulthood (30). This suggest that although the JIA-uveitis two group was on average older, the increase in memory B cell subsets in these cases is unlikely merely attributable to differences in age. Indeed, we also did not observe a correlation of memory B cell count and age in our cohort (Supplementary Figure S2).

In contrast, expansion of switched memory B cells has been linked to early onset of JIA presenting before age 6 years, which persisted throughout the disease course in these cases (29). The early onset of JIA is also a risk factor for uveitis, which would make it tempting to speculate that the early onset associated expansion of memory B cells is linked to uveitis development. In this small cohort, we did, however, not see a larger memory B cell gene fraction in early onset JIA (Supplementary Figure S3). This follows the work from Marasco and associates (29) who also did not find an association between JIA early onset related memory B cell expansion and uveitis. Our data suggest that this is the result of heterogeneity in the B cell compartment of JIA cases with uveitis. Although underpowered, we found suggestive evidence that cases with increased memory B cell signatures can be characterized by late-onset of uveitis (Supplementary Figure S3). We recommend further investigation into memory B cells in late-onset and early onset uveitis in cases with JIA to dissect the contribution of these immune cells in the pathophysiology of uveitis in JIA.

Some cases in this study used methotrexate. Although previous studies have shown that IgG-positive memory B cell frequency is increased in JIA patients treated with methotrexate, the absolute numbers of memory B cells are not affected (38), nor did methotrexate halt the progressive increase of memory B cells in early onset JIA (29). Also here, we would like to emphasize that conclusive data on changes in the composition of B cell subsets warrants further in-depth cytometry analysis ideally at different time points in cohorts of JIA cases with significant ophthalmological follow-up. We propose that such future analyses are accompanied by single-cell sequencing approaches of peripheral blood immune cells or iris biopsies of JIA cases with uveitis.

To date, several studies support a role for B cells in the pathogenesis of JIA-U+. Small studies have revealed increased abundance of CD20+ B cells in eye tissues of cases with JIA-uveitis. In fact, plasma cells and CD20^+^ B cells outnumbered other immune cells in enucleated eyes and iridectomy specimens (10, 11). A more recent study performed transcriptomic and proteomic analysis of iris tissue in three JIA-U+ and three primary open-angle glaucoma patients and detected increased expression for immunoglobulin genes and B cell-associated proteins in JIA-U+ (39). None of the upregulated B-cell associated genes identified in this study were found to be different between the disease groups in our study. This could suggests discrepancies between peripheral blood and primary site of inflammation, however, both studies support a role for memory B cells in JIA-associated uveitis. Although the mechanisms of B cell mediated immune responses in JIA-U remain unknown, intraocular antibodies against Parvovirus B19 were significantly higher in JIA-U + patients compared to children with other types of anterior uveitis, which may have triggered an immune response (40).

In summary, we found no differences in transcriptome of the peripheral blood CD19^+^ B cells pool in JIA-U- and JIA-U+ patients. However, dimension reduction-based analysis of B cell subset genes identified a distinct subset of JIA cases with uveitis characterized by the expression of memory B cell genes.

## Data Availability Statement

The original contributions presented in the study are included in the article/Supplementary Material, further inquiries can be directed to the corresponding author/s.

## Ethics Statement

The studies involving human participants were reviewed and approved by the Medical Ethical Research Committee in Utrecht. Written informed consent to participate in this study was provided by the participants’ legal guardian/next of kin.

## Author Contributions

RW: execution and interpretation of experiments and results, analysis of the data, and drafting the manuscript. AP: execution and interpretation of experiments and results, analysis of the data, and revising the manuscript. AMH, NW, and BV: acquisition of data and revising the manuscript. SH: acquisition of data, execution and interpretation of experiments and results, and revising the manuscript. VK: interpretation of results and revising the manuscript. TR study design, interpretation of experiments and results, and revising the manuscript. JB study design, interpretation of experiments and result, supervisory support, and revising the manuscript. JK study design, analysis of the data, interpretation of experiments and result, supervisory support, and drafting and revising the manuscript. All authors contributed to the article and approved the submitted version.

## Conflict of Interest

The authors declare that the research was conducted in the absence of any commercial or financial relationships that could be construed as a potential conflict of interest. The handling editor declared a past co-authorship with one of the author TR.
