# Supplementary figures and images for: Whole Transcriptome Analysis Reveals Heterogeneity in B Cell Memory Populations in Patients With Juvenile Idiopathic Arthritis-Associated Uveitis
Source: Front Immunol. 2020 Sep 17;11:2170. doi: 10.3389/fimmu.2020.02170 (PMC7527539; doi:10.3389/fimmu.2020.02170)

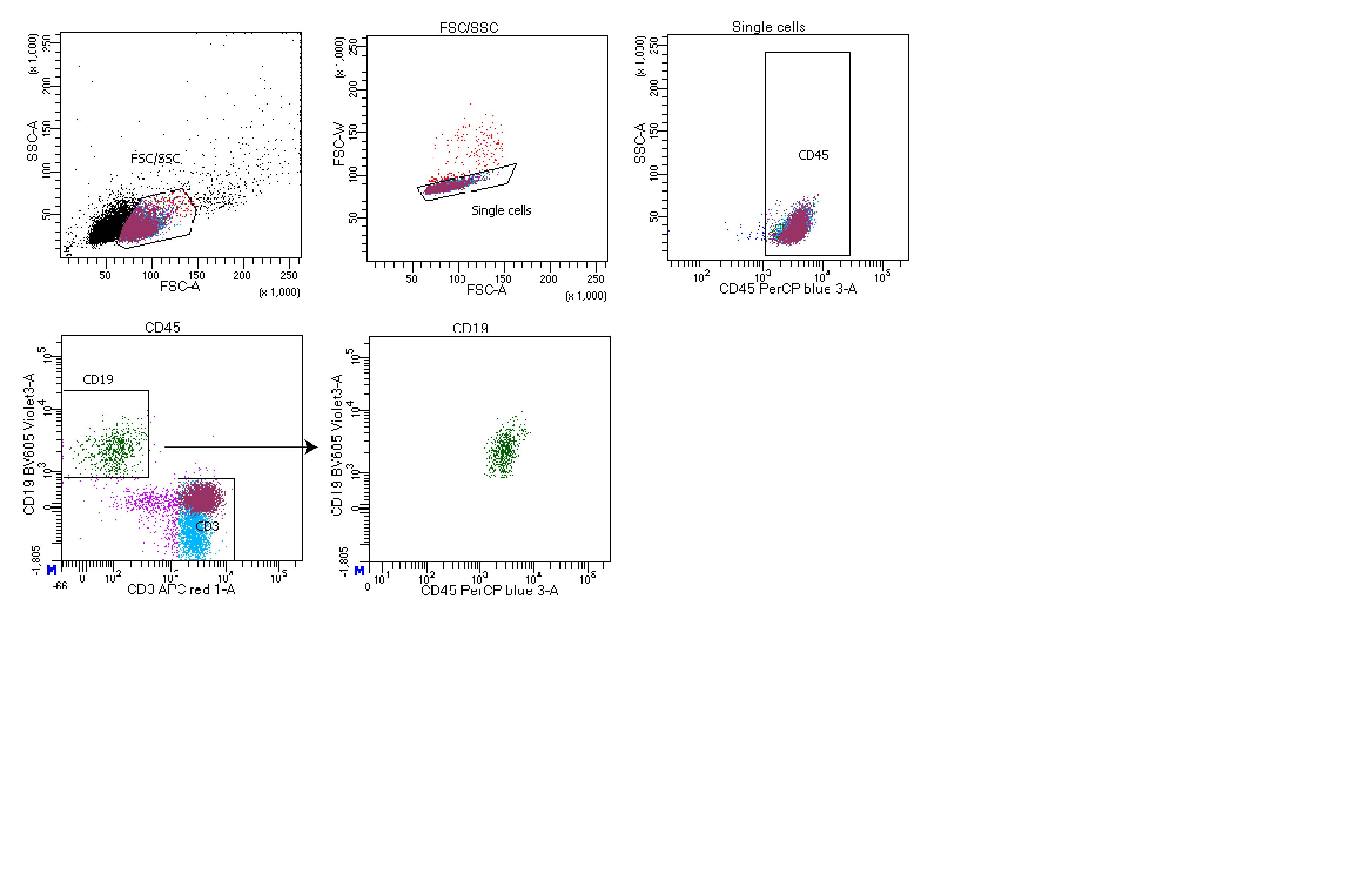

Supplement: Supplementary file 1 [file Image_1.jpeg]

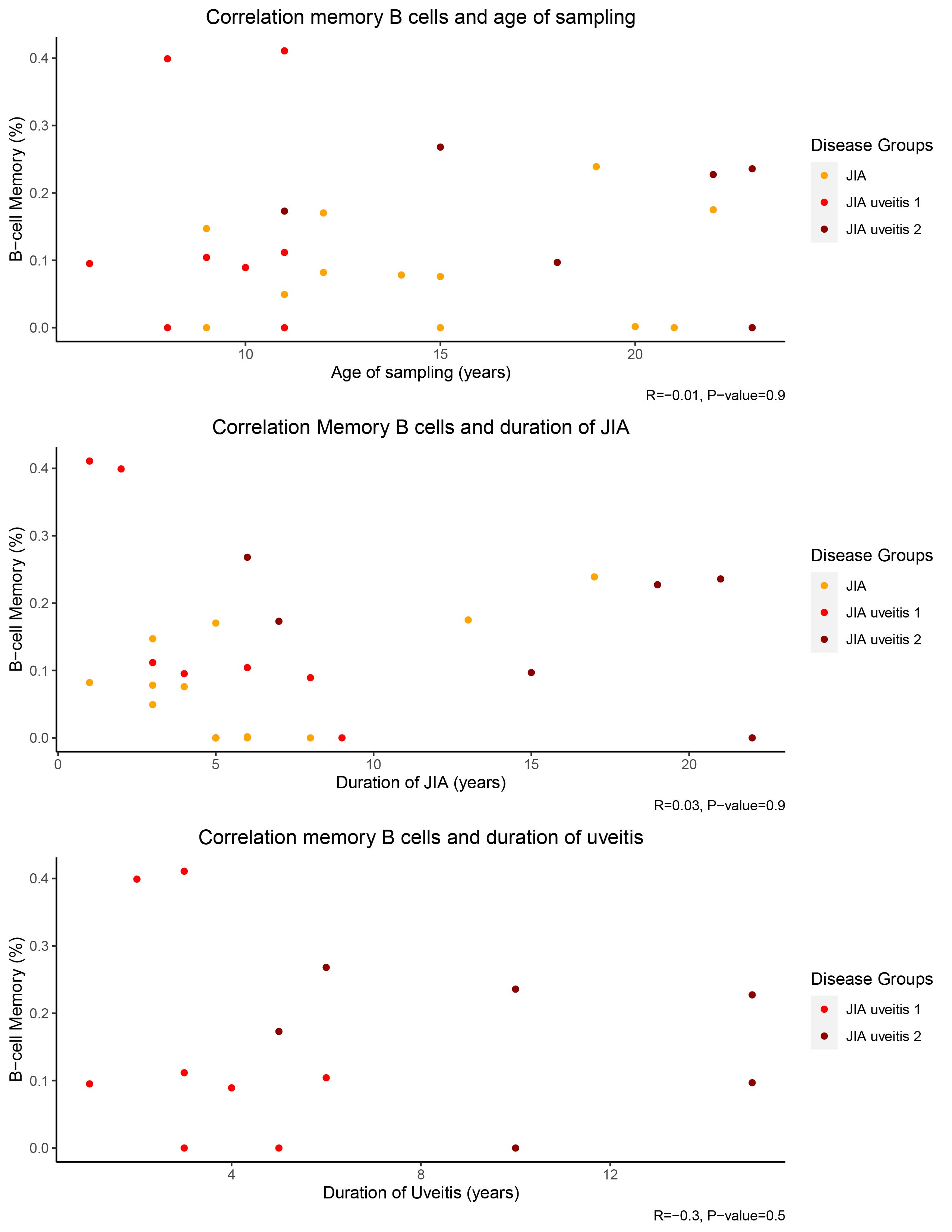

Supplement: Supplementary file 2 [file Image_2.JPEG]

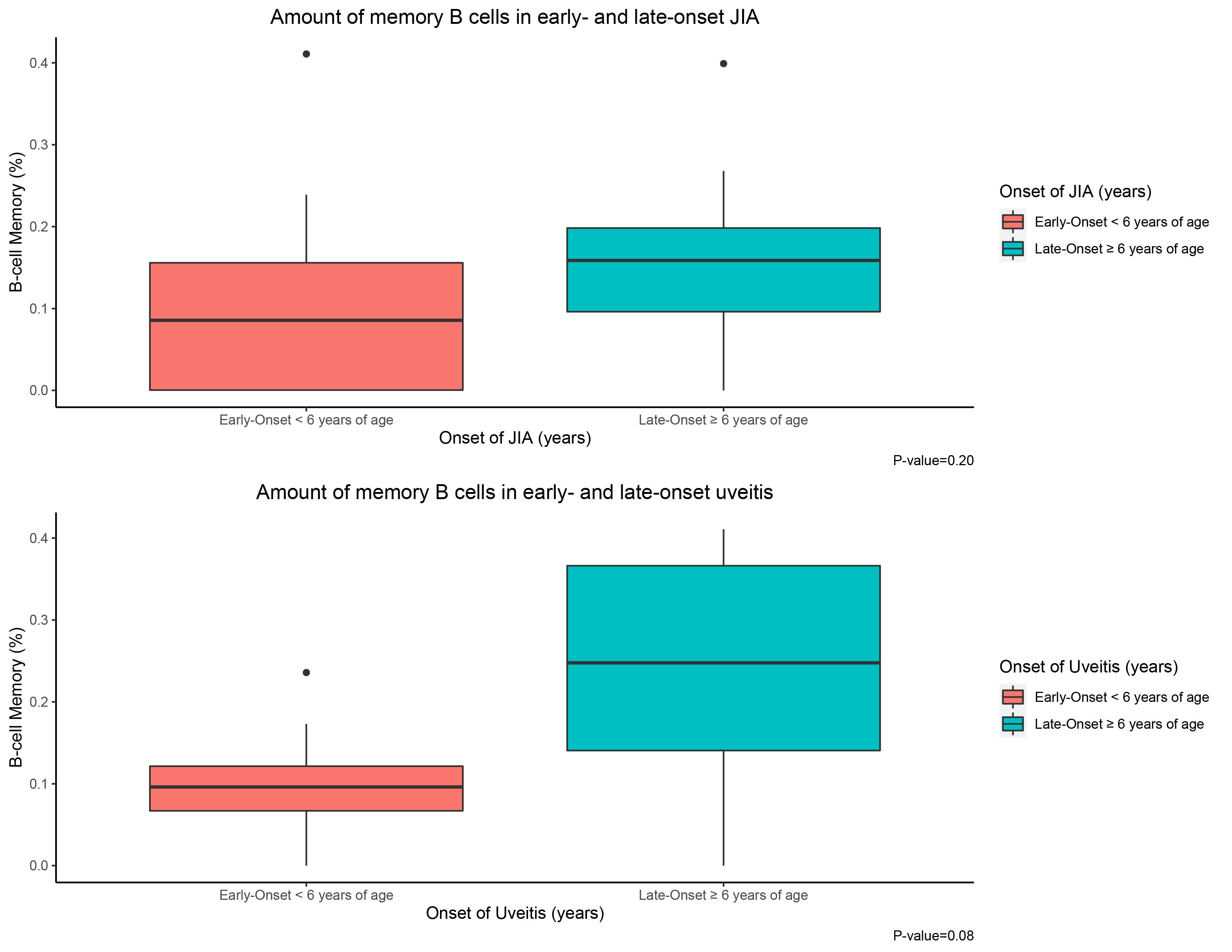

Supplement: Supplementary file 3 [file Image_3.jpeg]
